# Supplementary material for: A Naturally Occurring Microhomology-Mediated Deletion of Three Genes in African Swine Fever Virus Isolated from Two Sardinian Wild Boars
Source: Viruses. 2022 Nov 14;14(11):2524. doi: 10.3390/v14112524 (PMC9693351; doi:10.3390/v14112524)
Supplement: Supplementary file 1 [file viruses-14-02524-s001.zip › viruses-1978967-supplementary.pdf]

Table S1: List of primers used in this study for deletion detection.

Forward		Reverse		Start	Stop	Estimated Size
ASFV1-DEL_F	CAGACGTTGCCTATTCGG	ASFV1-DEL_R	GCTGAGAGACAATTTGCG	11386	12074	688
ASFV2-DEL_F	CCGCAAATTGTCTCTCAG	ASFV2-DEL_R	GCTCTGACGTTGACAGCT	12056	12994	938
ASFV3-DEL_F	GTCTTTCGTCCTTTCCG	ASFV3-DEL_R	GGACACCAGTGAACCTG	12866	12315	551
ASFV4-DEL_F	GCTGTCAACGTCAGAGCA	ASFV4-DEL_R	CACCAGTGAACCTGTTTC	12964	13294	330
ASFV5-DEL_F	AAACAGGTTCACTGGTGTC	ASFV5-DEL_R	GTCCAGATAAGCCTGACAT	13294	14241	947
ASFV6-DEL_F	GATGTCAGGCTTATCTGGA	ASFV6-DEL_R	ACGGACGTTGTTATCCTGG	14222	15042	820
ASFV7-DEL_F	GCTCCCCAGGATAACAAC	ASFV7-DEL_R	GCCATTGCTTTAGCATCT	15019	15569	550
ASFV8-DEL_F	CTGTACAGATGCTAAAGCAA	ASFV8-DEL_R	ACATATTTCACATCCGTGGC	15546	16400	854
ASFVfullDEL_F	CAGACGTTGCCTATTCGG	ASFVfullDEL_R	ACATATTTCACATCCGTGGC	11386	16400	5014

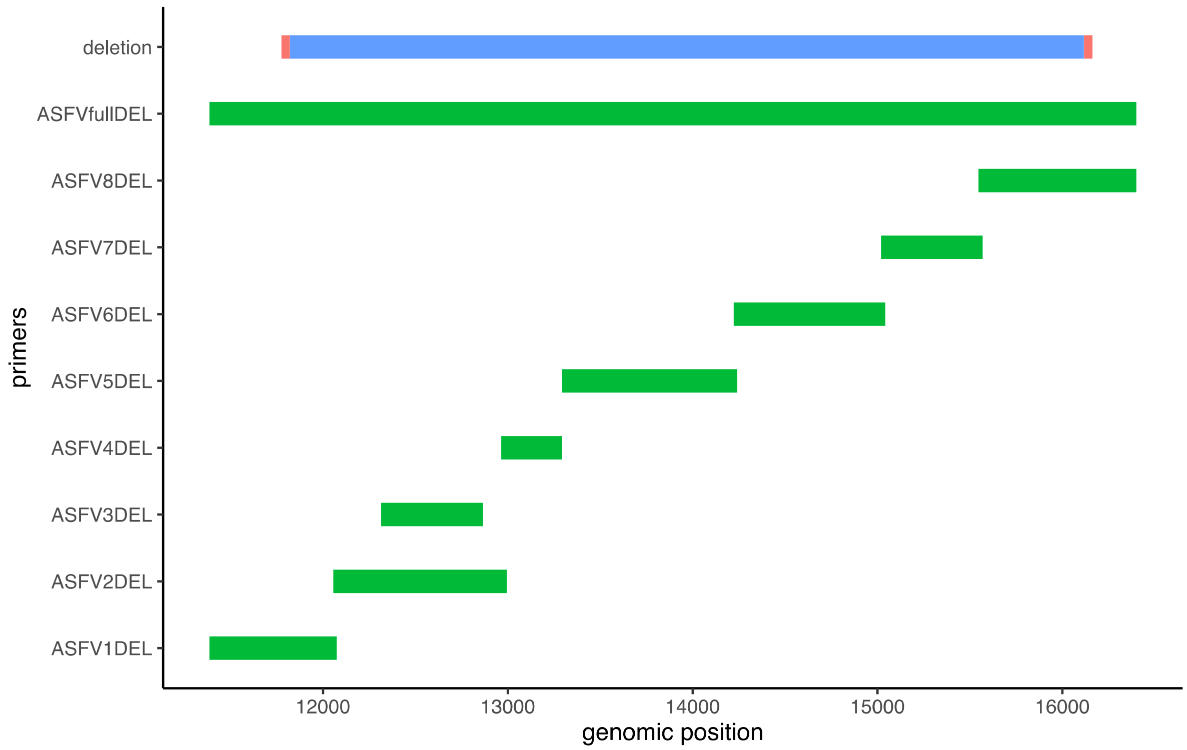

Figure S1. Primer locations along the ASFV genome with respect to the reference sequence KX354450. The choice of primers (green bars) was driven by the putative location of the deletion. The location of the deletion (blue bar) and of the regions of perfect microhomology (in red) is shown at the top.

Table S2: Metadata associated with ASFV whole genomes analyzed in this study. GenBank accession number and tip labels used in the phylogenetic trees are also reported.

SeqID	SeqName	SampleID	GenBank	Host	Sampling	Country	City
PRT|LISB|1960	PRT|LISB|1960	L60	NC044941	Domestic Pig	1960	Portugal	Lisbon
ESP|1975	ESP|1975	E75	NC044958	Domestic Pig	1975	Spain	
ITA|CA|1|1978	ITA|CA|1|1978	56/Ca/1978	MN270969	Domestic Pig	1978	Italy	Cagliari
ITA|CA|2|1978	ITA|CA|2|1978	CA1978_2	MW723480	Domestic Pig	1978	Italy	Cagliari
ITA|NU|1979	ITA|NU|1979	NU1979	MW723481	Domestic Pig	1979	Italy	Nuoro
ITA|CA|1979	ITA|CA|1979	57/Ca/1979	MN270970	Domestic Pig	1979	Italy	Cagliari
ITA|SS|1981	ITA|SS|1981	SS1981	MW788409	Domestic Pig	1981	Italy	Sassari
ITA|NU|1|1981	ITA|NU|1|1981	139/Nu/1981	MN270971	Domestic Pig	1981	Italy	Sassari
ITA|NU|2|1981	ITA|NU|2|1981	NU1981_2	SRR13976567	Domestic Pig	1981	Italy	Nuoro
ITA|TO|1983	ITA|TO|1983	ITALY1983	SRR13976568	Domestic Pig	1983	Italy	Torino
ITA|OR|1984	ITA|OR|1984	OR1984	MW800838	Domestic Pig	1984	Italy	Oristano
ITA|OR|1985	ITA|OR|1985	140/Or/1985	MN270972	Domestic Pig	1985	Italy	Oristano
ITA|CA|1985	ITA|CA|1985	85/Ca/1985	MN270973	Domestic Pig	1985	Italy	Cagliari
ITA|NU|1986	ITA|NU|1986	NU1986	MW723482	Domestic Pig	1986	Italy	Nuoro
ITA|NU|1|1990	ITA|NU|1|1990	NU1990_1	MW723483	Domestic Pig	1990	Italy	Nuoro
ITA|NU|2|1990	ITA|NU|2|1990	NU1990_2	SRR13976561	Domestic Pig	1990	Italy	Nuoro
ITA|NU|3|1990	ITA|NU|3|1990	141/Nu/1990	MN270974	Domestic Pig	1990	Italy	Nuoro
ITA|NU|2|1991	ITA|NU|2|1991	NU1991_2	MW723484	Domestic Pig	1991	Italy	Nuoro
ITA|NU|3|1991	ITA|NU|3|1991	NU1991_3	MW723485	Domestic Pig	1991	Italy	Nuoro
ITA|NU|7|1991	ITA|NU|7|1991	NU1991_7	MW723486	Domestic Pig	1991	Italy	Nuoro
ITA|NU|9|1991	ITA|NU|9|1991	NU1991_9	SRR13976566	Domestic Pig	1991	Italy	Nuoro
ITA|NU|1993-07-1	ITA|NU|Jul-1993	NU1993_2	MW723488	Domestic Pig	01/07/93	Italy	Nuoro
ITA|OR|1993	ITA|OR|1993	OR1993_1	MW723487	Domestic Pig	1993	Italy	Oristano
ITA|NU|1|1995	ITA|NU|1|1995	142/Nu/1995	MN270975	Domestic Pig	1995	Italy	Nuoro
ITA|NU|2|1995	ITA|NU|2|1995	NU1995_2	MW723489	Domestic Pig	1995	Italy	Nuoro
ITA|NU|3|1995	ITA|NU|3|1995	NU1995_3	MW723490	Domestic Pig	1995	Italy	Nuoro
ITA|NU|4|1995	ITA|NU|4|1995	NU1995_4	MW723491	Domestic Pig	1995	Italy	Nuoro
BEN|1997	BEN|1997	BENIN_1997	NC044956	Domestic Pig	1997	Benin	
ITA|TEM|2002-04-19	ITA|TEM|Apr-2002	24225_2002	MW788411	Domestic Pig	19/04/02	Italy	Tempio
ITA|ORU|2004-07-7	ITA|ORU|Jul-2004	44076_2004	MW723500	Domestic Pig	07/07/04	Italy	Orune
ITA|MAC|2004-07-12	ITA|MAC|Jul-2004	45539_2004	SRR13976563	Domestic Pig	12/07/04	Italy	Macomer
ITA|BUL|2004-10-5	ITA|BUL|Oct-2004	26/Ss/2004	MN270977	Domestic Pig	05/10/04	Italy	Bultei
ITA|TEL|2004-11-26	ITA|TEL|Nov-2004	74377_2004	MW723496	Domestic Pig	26/11/04	Italy	Telti

ITA丨SAD丨2005-05-5	ITA丨SAD丨May-2005	22649_2005	MW723497	Domestic Pig	05/05/05	Italy	Sadali
ITA丨PAL丨2005-12-1	ITA丨PAL丨Dec-2005	72398WB_2005	MW723495	Wild boar	01/12/05	Italy	Palau
ITA丨TER丨2007-11-23	ITA丨TER丨Nov-2007	72407_2007	MN270978	Domestic Pig	23/11/07	Italy	Tergu
ITA丨URZ丨2007-03-11	ITA丨URZ丨Mar-2007	72912WB_2007	MW723498	Wild boar	11/03/07	Italy	Urzulei
ITA丨URZ丨2008-01-6	ITA丨URZ丨Jan-2008	1537WB_2008	MW788405	Wild boar	06/01/08	Italy	Urzulei
ITA丨TAL丨2008-01-20	ITA丨TAL丨Jan-2008	4996WB_2008	MW723492	Wild boar	20/01/08	Italy	Talana
ITA丨BEN丨2008-04-10	ITA丨BEN丨1丨Apr-2008	22137_2008	MW723499	Domestic Pig	10/04/08	Italy	Benetutti
ITA丨VIL丨2008-04-16	ITA丨VIL丨1丨Apr-2008	22943_2008	MW788406	Domestic Pig	16/04/08	Italy	Villasor
ITA丨VIL丨2008-04-17	ITA丨VIL丨2丨Apr-2008	23221_2008	MW723494	Domestic Pig	17/04/08	Italy	Villasor
ITA丨BEN丨2008-04-28	ITA丨BEN丨2丨Apr-2008	25185_2008	MW788410	Domestic Pig	28/04/08	Italy	Benetutti
ITA丨STI丨2008-06-1	ITA丨STI丨Jun-2008	46830_2008	MW723493	Domestic Pig	01/06/08	Italy	Stintino
ITA丨STI丨2008-09-1	ITA丨STI丨Sep-2008	47/Ss/2008	KX354450	Domestic Pig	01/09/08	Italy	Stintino
ITA丨ORU丨2009-01-15	ITA丨ORU丨Jan-2009	1628_2009	SRR14601691	Wild boar	15/01/19	Italy	Orune
ITA丨ONI丨2009-05-22	ITA丨ONI丨May-2009	28170_2009	SRR13976565	Domestic Pig	22/05/09	Italy	Oniferi
ITA丨BAU丨2010-05-20	ITA丨BAU丨May-2010	26544/OG10	KM102979	Domestic Pig	20/05/10	Italy	Baunei
ITA丨BONO丨2011-06-14	ITA丨BONO丨Jun-2011	31208_2011	MW736612	Domestic Pig	14/06/11	Italy	Bono
ITA丨ALA丨2012-02-12	ITA丨ALA丨Feb-2012	2019WB_2012	MW736598	Wild boar	12/02/12	Italy	Ala dei Sardi
ITA丨OSC丨2012-04-18	ITA丨OSC丨Apr-2012	97/Ot/2012	MN270979	Domestic Pig	18/04/12	Italy	Oschiri
ITA丨BIT丨2011-12-30	ITA丨BIT丨Dec-2011	63525WB_2011	MW736603	Wild boar	30/12/11	Italy	Bitti
ITA丨BUD丨2013-03-25	ITA丨BUD丨Mar-2013	30322_2013	MW736600	Domestic Pig	25/03/13	Italy	Budduso
ITA丨BON丨2013-04-3	ITA丨BON丨Apr-2013	32516_2013	MW736607	Domestic Pig	03/04/13	Italy	Bonorva
ITA丨PAT丨2013-05-20	ITA丨PAT丨May-2013	47039_2013	MW736597	Domestic Pig	20/05/13	Italy	Pattada
ITA丨BOL丨2013-05-27	ITA丨BOL丨May-2013	49179WB_2013	MW736601	Wild boar	27/05/13	Italy	Bolotana
ITA丨PAT丨2013-11-11	ITA丨PAT丨Nov-2013	98039_2013	MW736599	Domestic Pig	11/11/13	Italy	Pattada
ITA丨NUL丨2013-12-16	ITA丨NUL丨Dec-2013	113049WB_2013	MW736608	Wild boar	16/12/13	Italy	Nulvi
ITA丨CAS丨2014-01-27	ITA丨CAS丨Jan-2014	11484WB_2014	SRR13975654	Wild boar	27/01/14	Italy	Castelsardo
ITA丨VILLA丨2014-02-21	ITA丨VILLA丨Feb-2014	22653/Ca/2014	MN270980	Domestic Pig	21/02/14	Italy	Villanovatulo
ITA丨BON丨2014-03-31	ITA丨BON丨Mar-2014	35479_2014	MW788408	Domestic Pig	31/03/14	Italy	Bonorva
ITA丨TER丨2014-06-3	ITA丨TER丨Jun-2014	51268_2014	MW736605	Domestic Pig	03/06/14	Italy	Tergu
ITA丨BEN丨2015-01-14	ITA丨BEN丨Jan-2015	6396WB_2015	MW736609	Wild boar	14/01/15	Italy	Benetutti
ITA丨ORG丨2015-02-10	ITA丨ORG丨Feb-2015	15998_2015	MW736604	Domestic Pig	10/02/15	Italy	Orgosolo
ITA丨BON丨2015-03-25	ITA丨BON丨Mar-2015	28928_2015	MW736610	Domestic Pig	25/03/15	Italy	Bonorva
ITA丨SEN丨2015-04-10	ITA丨SEN丨Apr-2015	31479_2015	MW788407	Domestic Pig	10/04/15	Italy	Sennori
ITA丨ANE丨2015-04-21	ITA丨ANE丨Apr-2015	33747WB_2015	MW736613	Wild boar	21/04/15	Italy	Anela
ITA丨SAR丨2016-06-16	ITA丨SAR丨Jun-2016	53706_2016	MW736602	Domestic Pig	16/06/16	Italy	Sarule

ITA|DOL|2017-01-10	ITA|DOL|Jan-2017	3312_2017	SRR13976564	Domestic Pig	10/01/17	Italy	Dolianova
ITA|DES|2017-03-14	ITA|DES|Mar-2017	34403WB_2017	MW736606	Wild boar	14/03/17	Italy	Desulo
ITA|SEUI|2017-05-23	ITA|SEUI|May-2017	52060_2018	SRR13976569	Free Ranging Pig	23/05/17	Italy	Seui
ITA|ARI|2018-01-15	ITA|ARI|Jan-2018	8343_2018	SRR13976571	Free Ranging Pig	15/01/18	Italy	Aritzo
ITA|LOT|2018-06-8	ITA|LOT|1|Jun-2018	54684_2018	MW647171	Free Ranging Pig	08/06/18	Italy	Lotzorai
ITA|LOT|2018-06-14	ITA|LOT|2|Jun-2018	56140_2018	MW736611	Free Ranging Pig	14/06/18	Italy	Lotzorai
ITA|DES|2018-06-15	ITA|DES|Jun-2018	55234_2018	MT932579	Free Ranging Pig	11/06/18	Italy	Desulo
ITA|TAL|2018-12-15	ITA|TAL|Dec-2018	103917_2018	MT932578	Free Ranging Pig	17/12/18	Italy	Talana
ITA|LAN|2019-01-14	ITA|LAN|Jan-2019	7212_2019	ON260838	Wild boar	14/01/19	Italy	Lanusei
ITA|PAT|2019-01-14	ITA|PAT|Jan-2019	7303_2019	ON260839	Wild boar	13/01/19	Italy	Pattada

Table S3: Metadata associated with ASFV microhomology analysis.

Sequence name	Country	Genotype
KX354450.1 African swine fever virus isolate 47/Ss/2008, complete genome	Italy	I
MN270969.1 African swine fever virus isolate 56/Ca/1978, complete genome	Italy	I
NC_044958.1 African swine fever virus E75 complete genome, strain E75	Spain	I
NC_044941.1 African swine fever virus strain L60, complete genome	Portugal	I
MN913970.1 African swine fever virus strain Liv13/33 (OmLF2) isolate OmLF2, complete genome	France	I
NC_044957.1 African swine fever virus OURT 88/3 (avirulent field isolate), complete genome	Portugal	I
U18466.2 African swine fever virus strain BA71V, complete genome	Spain	I
NC_044943.1 African swine fever virus strain NHV, complete genome	Portugal	I
NC_044959.2 African swine fever virus isolate ASFV Georgia 2007/1 genome assembly, complete genome: monopartite	Georgia	II
AY261365.1 African swine fever virus isolate Warmbaths, complete genome	South Africa	III
MN641876.2 African swine fever virus isolate RSA_W1_1999, complete genome	South Africa	IV
AY261361.1 African swine fever virus isolate Malawi Lil-20/1 (1983), complete genome	Malawi	VIII
MH025916.1 African swine fever virus strain R8, complete genome	Uganda	IX
MW856067.1 African swine fever virus strain BUR/18/Rutana, complete genome	Burundi	X
MN641877.2 African swine fever virus isolate RSA_2_2004, complete genome	South Africa	XX
MN336500.3 African swine fever virus isolate RSA_2_2008, complete genome	South Africa	XXII

Table S4: Deletions and changes between the two isolates under study with the reference strain KX354450. Genome position, nucleotide, type of mutation, and predicted features are reported.

Gene	Genome Position	Ref	Alt_7212WB/19	Alt_7303WB/19	Aa_chg	Mutation	Predicted features
IG	11820..12052	no deletion	deletion	deletion			Intergenic region
MGF360-6L	12053..13180	no deletion	deletion	deletion			MGF360 family
IG	13181..14063	no deletion	deletion	deletion			Intergenic region
X69R	14064..14273	no deletion	deletion	deletion			Uncharacterized protein
IG	14274..14459	no deletion	deletion	deletion			Intergenic region

MGF300-1L	14460..15266	no deletion	deletion	deletion			MGF300 family
IG	15267..16162	no deletion	deletion	deletion			Intergenic region
MGF 505-3R (*)	30649	A	G	G	Silent	Point mutation	MGF505 family
IG (*)	34552	T	C	C	NA	Point mutation	Intergenic region
MGF360-15R (*)	45961	T(7)	T(8)	T(8)	Frameshift	INDEL	MGF360 family
URF19 (*)	54920	C	T	T	Y21C	Point mutation	Undefined reading frame (Hypothetical protein)
EP364R (*)	71175	A	G	G	A319T	Point mutation	XPF_NUCLEASE-LIKE
EP364R (*)	71216	A	G	G	SIlent	Point mutation	XPF_NUCLEASE-LIKE
URF22 (*)	77296	A(9)	A(10)	A(10)	Frameshift	INDEL	Undefined reading frame (Hypothetical protein)
C315R (*)	83384	T	C	C	Silent	Point mutation	TFIIB-like factor
IG (*)	95173	G	A	A	NA	NA	Intergenic region
IG (*)	110196	G	A	A	NA	NA	Intergenic region
D205R (*)	140311	G	A	A	P131S	Point mutation	RNA polymerase subunit 5
S273R	142703	G	A	A	Silent		Cysteine protease S273R
URF56 (*)	152555	G(5)	G(6)	G(6)	Frameshift		Undefined reading frame (Hypothetical protein)
IG (*)	176705	A(7)	A(8)	A(8)	Na		Intergenic region
IG (*)	179838	C(9)	C(10)	C(10)	Na		Intergenic region

(*): changes in Sardinian ASFV isolates already described [16,50]

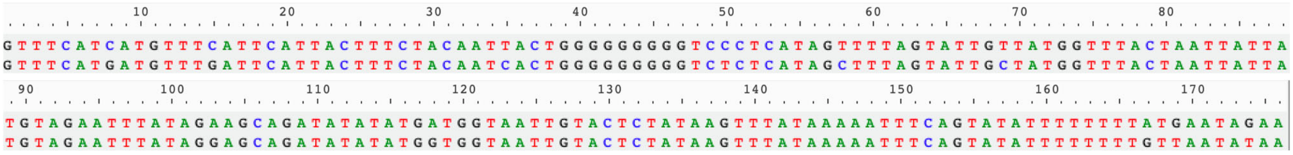

Figure S2. Alignment of the two regions of imperfect microhomology starting at positions 11656 and 15998 of the Sardinian ASFV reference sequence (KX354450).

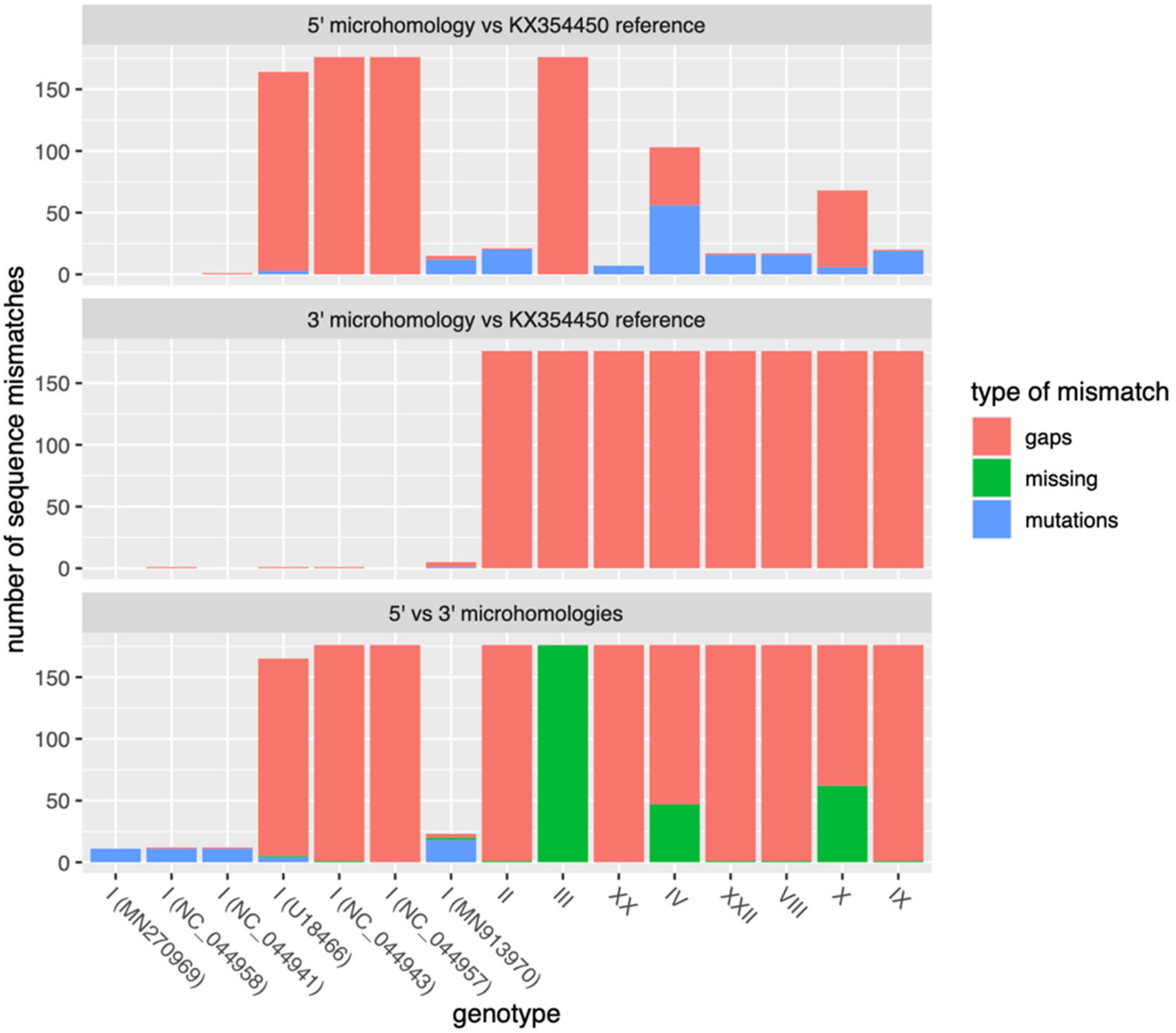

Figure S3. Conservation of the imperfect microhomology and the two 176 bp sequences involved (one at the 5′ end of the deletion, one at the 3′) among ASFV genotypes. Top: number of mismatches between the sequence closer to the 5' end of the genome in KX354450 and the corresponding sequences in other samples from different genotypes, ordered by genomic divergence from KX354450. Middle: mismatches between the sequence closer to the 3' end of the genome in KX354450 and the corresponding sequences in other samples from different genotypes. Bottom: mismatches between the two sequences from the same sample. "Missing" denotes bases that are missing (i.e. contain gaps in the alignment) for both sequences.
